# Tousled-Like Kinase-Dependent Phosphorylation of Rad9 Plays a Role in Cell Cycle Progression and G2/M Checkpoint Exit

**DOI:** 10.1371/journal.pone.0085859

**Published:** 2013-12-20

**Authors:** Ryan Kelly, Scott K. Davey

**Affiliations:** 1 Division of Cancer Biology and Genetics, Cancer Research Institute, Queen’s University, Kingston, Ontario, Canada; 2 Department of Biomedical and Molecular Sciences, Queen’s University, Kingston, Ontario, Canada; 3 Department of Pathology and Molecular Medicine, Queen’s University, Kingston, Ontario, Canada; University of Hawaii Cancer Center, United States of America

## Abstract

Genomic integrity is preserved by checkpoints, which act to delay cell cycle progression in the presence of DNA damage or replication stress. The heterotrimeric Rad9-Rad1-Hus1 (9-1-1) complex is a PCNA-like clamp that is loaded onto DNA at structures resulting from damage and is important for initiating and maintaining the checkpoint response. Rad9 possesses a C-terminal tail that is phosphorylated constitutively and in response to cell cycle position and DNA damage. Previous studies have identified tousled-like kinase 1 (TLK1) as a kinase that may modify Rad9. Here we show that Rad9 is phosphorylated in a TLK-dependent manner *in vitro* and *in vivo*, and that T355 within the C-terminal tail is the primary targeted residue. Phosphorylation of Rad9 at T355 is quickly reduced upon exposure to ionizing radiation before returning to baseline later in the damage response. We also show that TLK1 and Rad9 interact constitutively, and that this interaction is enhanced in chromatin-bound Rad9 at later stages of the damage response. Furthermore, we demonstrate via siRNA-mediated depletion that TLK1 is required for progression through S-phase in normally cycling cells, and that cells lacking TLK1 display a prolonged G2/M arrest upon exposure to ionizing radiation, a phenotype that is mimicked by over-expression of a Rad9-T355A mutant. Given that TLK1 has previously been shown to be transiently inactivated upon phosphorylation by Chk1 in response to DNA damage, we propose that TLK1 and Chk1 act in concert to modulate the phosphorylation status of Rad9, which in turn serves to regulate the DNA damage response.

## Introduction

 Cell cycle checkpoints comprise an elaborate network of signal transduction pathways that maintain the proper order of cellular events [1]. Checkpoints can initiate a delay in progression through the cell cycle in response to both endogenous and exogenous DNA damage, thus granting the cell time to repair damage and ensuring that damaged DNA is not replicated and passed on to subsequent generations [2,3]. The importance of proper checkpoint function is underscored by the fact that mutations in checkpoint genes can lead to genetic instability and are found in a host of human cancers and cancer predisposition syndromes [4–8].

 The human 9-1-1 complex is a proliferating cell nuclear antigen (PCNA)- like heterotrimeric DNA clamp composed of Rad9, Rad1, and Hus1 [9–11]. The 9-1-1 complex is loaded onto DNA at 5’-recessed ends, a common substrate resulting from DNA metabolism and damage [12–14]. Chromatin-bound 9-1-1 is thought to act as a scaffold that localizes other elements of the checkpoint machinery to sites of DNA damage and thus plays a critical role in initiating and maintaining the checkpoint response [15,16]. In particular, the 9-1-1 complex recruits DNA topoisomerase 2-binding protein 1 (TopBP1) to damage-induced lesions, thus facilitating activation of the PI3K-related kinase ataxia telangiactasia and Rad3-related (ATR) [17–19]. The 9-1-1 – TopBP1 – ATR module is required for efficient activation of Chk1 [20], a checkpoint kinase that inhibits Cdc25 phosphatase activity and delays the transition from G2 to mitosis [21–23].

 Rad9 is unique among the other components of the 9-1-1 complex in that it possesses an unstructured C-terminal tail of approximately 110 amino acids that does not share homology with Rad1, Hus1 or PCNA [24–27]. This region is not required for 9-1-1 complex formation [11,28] but it is necessary for TopBP1 association and proper checkpoint function [15,17,29]. In addition, it is heavily modified by phosphorylation both constitutively and transiently in response to cell cycle position and DNA damage, and hence represents a potential regulatory mechanism for checkpoint control [15,30–32]. For example, S272 is phosphorylated rapidly and transiently in response to damage regardless of cell cycle position by the PI3K-related kinase ataxia telangiactasia-mutated (ATM) [15,33], T292 is targeted during mitosis by Cdc2 [30], and S341 and S387 are both phosphorylated constitutively and are required for the interaction between Rad9 and TopBP1 [15,31]. While our lab and others have made progress towards identifying the targeted residues within Rad9 and the context under which they are modified, our understanding is far from complete. The complex and interdependent nature of Rad9 phosphorylation has made the task of establishing the physiological significance of these events challenging. 

 The human Tousled-like kinases 1 & 2 (TLK1 and TLK2) are homologues of *Arabidopsis thaliana* Tousled, and they exhibit peak activity in S-phase and likely participate in chromatin remodeling [34,35]. TLK1 and TLK2 are thought to oligermerize [35], and both phosphorylate the H3/H4 histone chaperone anti-silencing function 1 homolog A (ASF1A), which itself facilitates histone deposition and chromatin assembly during S-phase and following DNA repair[36–39]. The physiological significance of TLK-dependent phosphorylation of ASF1A is poorly understood, although there is evidence that it may serve to protect it from proteasomal degradation [40]. TLK1 in particular is inactivated rapidly in response to double-stranded breaks via ATM and Chk1-dependent phosphorylation at S695, and thus represents a regulatory link between cell cycle progression and checkpoint function [41,42]. Direct Chk1-induced inhibition of TLK1 is transient, and TLK1 activity returns to baseline levels later in the damage response. A recent report suggested that Rad9 is a substrate of TLK1, and that S328 within the C-terminal tail is the targeted residue [43]. Given that the 9-1-1 complex is required for damage-induced Chk1 activation [17,29], we were intrigued by the notion that a substrate of Chk1 may regulate Rad9 and thereby fine-tune the checkpoint response. Thus, we sought to further characterize the relationship between Rad9 and TLK activity. 

In this study we show that Rad9 is subject to TLK-dependent phosphorylation at T355, and that this event represents part of a feedback loop that controls checkpoint function. Furthermore, our data suggest that the interaction between Rad9 and TLK1 plays a role in normal cell cycle progression and facilitates termination of the G2/M checkpoint.

## Materials and Methods

### Cell culture and transfections

HeLa cells (CCL-2), obtained from the ATCC repository (Manassas, VA), were maintained in Dulbecco’s modified Eagle’s Medium (DMEM; Sigma, St. Louis, MO) supplemented with 10% fetal bovine serum (FBS; Life Technologies, Burlington, ON) at 37°C in 5% C02 atmosphere. Transient DNA transfections were carried out using Fugene 6 (Roche, Mississauga, ON) according to the manufacturer’s protocol using a 3:1 Fugene/DNA ratio. Small-interfering RNA (siRNA) transfections were carried out in 6-well plates using 3μl of Lipofectamine 2000 (Life Technologies) and 40pmol of siRNA duplex per well. siRNA directed against TLK1 (AM-51333) and a non-silencing scrambled siRNA (AM-4611) were purchased from Life Technologies.

### Drug treatments and irradiation

Cells were exposed to IR using a Victoreen Electometer 137Cs γ-irradiator (Atomic Energy of Canada, Mississauga, ON) at 0.45Gy/min. Thymidine (BioShop, Burlington, ON) was administered at 2mM for 18hr. 2hr prior to subsequent treatment, cells were washed twice with 5mL phosphate-buffered saline (PBS) and released into fresh DMEM supplemented with 10% FBS. Hydroxyurea (HU, Sigma) was administered at 10mM for 18hr.

### Plasmids and site-directed mutagenesis

All Rad9 point-mutants were generated using the Transformer site-directed mutagenesis kit (Clontech, Mountain View, CA) according to the manufacturer’s instructions. Rad9 constructs transfected into HeLa cells were contained within the pyDF vector [30] under influence of the SR-α promoter. N-terminal GST-fusion expression plasmids were generated by PCR subcloning either full-length or segments of Rad9 cDNA (both wild-type and point-mutants) into the pGEX-2T vector. 

### Antibodies

Rabbit polyclonal α-Rad9 phospho-T355 was raised and purchased from Pacific Immunology (Ramona, CA). Affinity-purified chicken polyclonal α-Rad9 antibodies used for immunoprecipitation and immunofluorescence were produced as previously described [9]. Other antibodies employed in this study were mouse α-Rad9 (611324, BD Biosciences, Mississauga, Canada), rabbit α-Rad9 phospho-S272 (AP-3223, Abgent, San Diego CA), rabbit α-TLK1 (for immunoprecipitation: ab74551, Abcam, Toronto, ON), rabbit α-TLK1 (for immunoblotting: 4125-S), rabbit α-TLK1 phospho-S695 (4121-S), mouse α-Chk1 (2360-S), rabbit α-Chk1 phospho-S317 (2344-S, Cell Signaling, Danvers, MA), mouse α-c-myc 9E10 (sc-40, Santa Cruz Biotechnology, Paso Robles CA), and chicken α-GAPDH (15822-100, Abcam). 

### Immunoprecipitation and immunoblotting

Cells were lysed in NETN buffer (250mM NaCl, 20mM Tris pH 8.0, 0.5% Nonidet P-40 and 10% glycerol supplemented with 20mM β-glycerophosphate, 0.2mM sodium fluoride, 1mM sodium orthovandate and 100μl of HALT EDTA-free protease inhibitor cocktail {Thermo Scientific, Rockford, IL} per 1mL lysis buffer) at a concentration of 1mL per 1.0 x 10^6^ cells. Lysates were then incubated in the presence or absence of 100 units/mL DNase I (Thermo Scientific) for 30 min at RT, after which they were centrifuged for 15 min at 13,000 x g, 4°C. The protein concentration of the supernatants was assayed using the DC^TM^ protein assay kit (Bio-Rad, Mississauga, ON), after which they were equalized and pre-cleared with α-chicken IgY-agarose (Aves Labs, Tigard, OR) for 20min at 4°C prior to immunoprecipitation. Immunoprecipitation was performed by incubating lysates with 2.0μg chicken polyclonal α-Rad9 per 1.0mg lysate protein overnight at 4°C and 40μl of a 1:1 α-chicken IgY-agarose and NETN slurry for 3hr at 4°C. Immune complexes were washed three times with 800μl NETN and re-suspended in 30μl SDS-PAGE loading buffer. Immunoblotting was carried out as previously described [30]. Blots were developed using X-ray film (Santa Cruz Biotechnology) or an Image Station 4000mM Pro imager (Kodak, Rochester, NY). Densitometry was performed using Carestream Molecular Imaging software (Carestream Molecular Imaging, New Haven, CT). 

### Protein purification

GST-fusion peptide expression was induced in 100mL of logarithmically growing BL21 *E. coli* with 10μl of 1M isopropyl-1-thio-β-D-galactopyranoside (final concentration 0.1mM) for 3hr at 37°C. Bacteria were lysed with a Sonic Dismembrator 100 sonicator (Fisher, Pittsburgh, PA) using four 20s pulses with 30s gaps. GST-fusion peptides were purified batch-wise with glutathione-sepharose (GE Healthcare, Buckinghamshire, UK). Bound protein was eluted with buffer containing 20mM reduced glutathione in 10mM HEPES 10mM MgCl2, and 1mM dithiothreitol at a final pH of 7.4. Full-length GST-Rad9 was concentrated post-elution with Mircrocon centrifugal filters (Millipore, Etobicoke, ON). 

### In vitro kinase assays

Asynchronously growing HeLa cells or those exposed to 20Gy IR or 10mM HU were harvested 18hr post-treatment and lysed in 1mL of kinase lysis buffer (50mM Tris pH 7.4, 1mM EDTA pH 8.0, 25mM NaCl and 0.1% Nonidet P-40 supplemented with 20mM β-glycerophosphate, 2mM NaF, 1mM Na_3_VO_4_, and one Complete-Mini protease inhibitor tablet {Roche} per 10mL) per 1.0 x 10^6^ cells. TLK1 was immunoprecipitated from these lysates using 1.5μg of rabbit polyclonal antibody per 1.0mg of protein and pulled down using protein A-conjugated sepharose (BioVision, Burlington, ON). Recombinant myc-TLK1 was immunoprecipitated using 1.5μg of mouse monoclonal 9E10 c-myc antibody per 1.0mg of protein, and pulled down using protein G-conjugated sepharose (BioVision). Immune complexes were washed three times with 800μl kinase lysis buffer and twice with 800μl kinase reaction buffer (50mM HEPES pH 7.4, 10mM MgCl2, 1mM dithiothreitol, 50μM ATP). Kinase reactions were carried out as previously described [15]. Phosphorylation was quantified using a Storm 820 phospho-imager and ImageQuant software (Molecular Dynamics, Sunnyvale, CA). 

### Flow cytometry

For one-dimensional cell cycle experiments, HeLa cells were harvested by trypsynization and fixed with 70% ethanol in PBS containing 1% FBS from between 30min to overnight at -20°C. Cells were collected by centrifugation and re-suspended in PBS containing 50μg/mL propidium iodide (PI, Calbiochem, Etobicoke, ON) and 0.1mg/mL RNase A (BioShop), and analyzed using an FC-500 flow cytometer (Beckman-Coulter, Mississauga, ON). For dual-staining experiments, 10μl of cell proliferating and labeling reagent containing bromodeoxyuridine (BrdU, GE Healthcare) was added to cells 1hr prior to harvesting. After fixation, cells were treated with 0.5% Triton X-100-H2O and 4N HCl, and neutralized with 0.1M sodium tetraborate (pH 8.0). Cells were then incubated with 1.0μg of fluorescein isothiocyanate (FITC)-conjugated α-BrdU antibody (eBioscience, San Diego, CA) prior to staining with PI. For indirect immunoflourescence of Rad9, cells were fixed with 2% paraformaldehyde for 20min at room temperature and then washed twice with PBS. Cells were permeabilized and blocked by incubation with PBS containing 0.1% Triton X-100 and 5% normal goat serum (Life Technologies), and staining was carried out using 0.5μg chicken polyclonal α-Rad9 antibody and 0.5μg Alexa 488-conjugated goat α-chicken antibody (Life Technologies). All flow cytometric data was analyzed using FlowJo 7.6 (TreeStar, Ashland, OR).

## Results

### Rad9 T355 is a substrate for TLK-dependent phosphorylation

**Figure 1:**
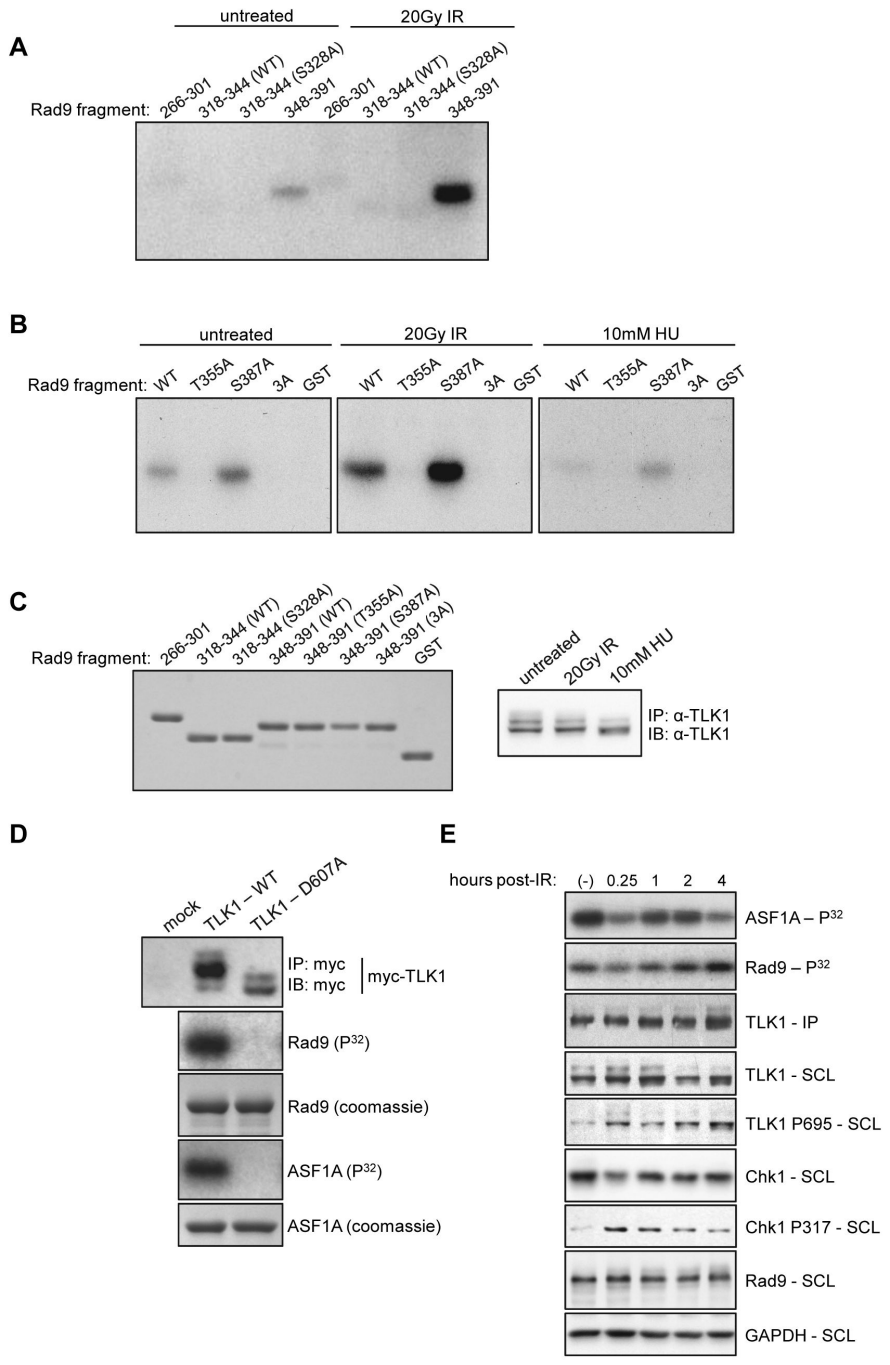
*In* *vitro* phosphorylation of Rad9 C-terminal fragments by TLK1. A & B. Recombinant GST-fusion peptides corresponding to different regions of Rad9’s C-terminal tail were employed as substrates for *in* *vitro* kinase assays using TLK1 immunoprecipitated from HeLa cells exposed to the indicated conditions. 3A refers to a T355A/S363A/S387A triple mutant. C. Substrate input (Rad9, left panel) and kinase input (TLK1, right panel) for each kinase reaction. IP refers to immunoprecipitate, IB refers to immunoblot. D. Overexpressed, recombinant WT and kinase-dead (D607A) myc-TLK1 constructs were immunoprecipitated from HeLa cells and employed in *in* *vitro* kinase assays using full-length GST-Rad9 and GST-ASF1a as substrates. E. A time course *in* *vitro* kinase assay. TLK1 was immunoprecipitated from HeLa cells exposed to 20Gy IR and harvested at the indicated time-points. Immune complexes were incubated with either recombinant GST-Rad9 (amino acids 348-391) or GST- ASF1A. SCL refers to soluble cell lysates. Images shown are representative of two independent experiments.

TLK1 plays a role in chromatin remodeling and S-phase progression [35,36], and is inhibited by Chk1-dependent phosphorylation at S695 in response to DNA damage [40–42]. A recent report [43] presented evidence that Rad9 was a substrate for TLK1, and that S328 was the targeted residue. Intrigued by the possibility of a Chk1-TLK1-Rad9 signaling axis that may regulate checkpoint signaling, we sought to further characterize the interaction between Rad9 and TLK1. To this end, we carried out *in vitro* kinase assays by incubating TLK1 immunoprecipitated from asynchronously growing HeLa cells or from cells harvested 18hr after exposure to 20Gy ionizing radiation (IR) with a panel of N-terminal GST-fusion peptides derived from Rad9’s heavily phosphorylated C-terminal tail. HeLa cells remain checkpoint arrested, and exhibit high levels of Rad9 phosphorylation at the 18h time point (30). Immunoprecipitated TLK1 phosphorylated the peptide fragment corresponding to Rad9 348-391 (Figure 1A), and phosphorylation increased when TLK1 was immunoprecipitated from cells that were pre-exposed to IR. Interestingly, there was only a background amount of phosphorylation present in the sample with the fragment containing S328 (318-344), and mutating S328 to alanine has no effect on TLK1-dependent phosphorylation (Figure 1A, lanes 6 & 7). Further analysis using additional peptides featuring point-mutations within the Rad9 348-391 fragment revealed that T355 of Rad9 was the only residue being modified (Figure 1B). Treatment with 10mM HU did not induce phosphorylation in the same manner as IR (Figure 1B), and in fact appeared to repress it. The input of both substrate and kinase in each reaction was consistent (Figure 1C, left and right panel respectively) and thus are not a factor in these results. To establish that Rad9 phosphorylation in this system was TLK1-direct, we performed additional *in vitro* kinase assays using recombinant, myc-tagged TLK1 and a kinase-dead myc-TLK1 harboring a D607A mutation within the kinase domain [35]. Plasmids encoding these constructs were transfected into HeLa cells, after which the kinases were immunoprecipitated using an antibody directed against the myc epitope. As shown in Figure 1D, kinase-dead myc-TLK1 was unable to phosphorylate full-length GST-Rad9 and GST-ASF1A, a well-characterized substrate of TLKs [36,40,42]. Thus, these results indicate that Rad9 phosphorylation in these *in vitro* assays was indeed TLK1-direct.

 Previous studies have shown that Chk1-dependent inhibition of TLK1 following irradiation is transient and gradually returns to a baseline level of activity [42]. We thus investigated whether TLK1 has a similar activity profile with regards to Rad9 by carrying out a time-course kinase assay. TLK1 was immunoprecipitated from cells that had been treated with 20Gy IR and harvested at progressive time-points post-damage, and was then incubated with the Rad9 peptide fragment containing T355 (348-391) or recombinant GST- ASF1A. A similar activity profile was observed for both ASF1A and Rad9 (Figure 1E). Phosphorylation of both ASF1A and Rad9 was reduced by 0.25hr post-IR, followed by a return to baseline levels by 2-4hr. This coincided with the rapid and transient activation of Chk1, as indicated by phosphorylation at S317. Finally, TLK1 phosphorylation at S695 persisted as Chk1 activity waned and ASF1A and Rad9 phosphorylation returned to baseline levels, which may indicate that other phosphatases or regulatory elements are responsible for TLK1 dephosphorylation, in addition to a decrease in Chk1 activity. 

**Figure 2:**
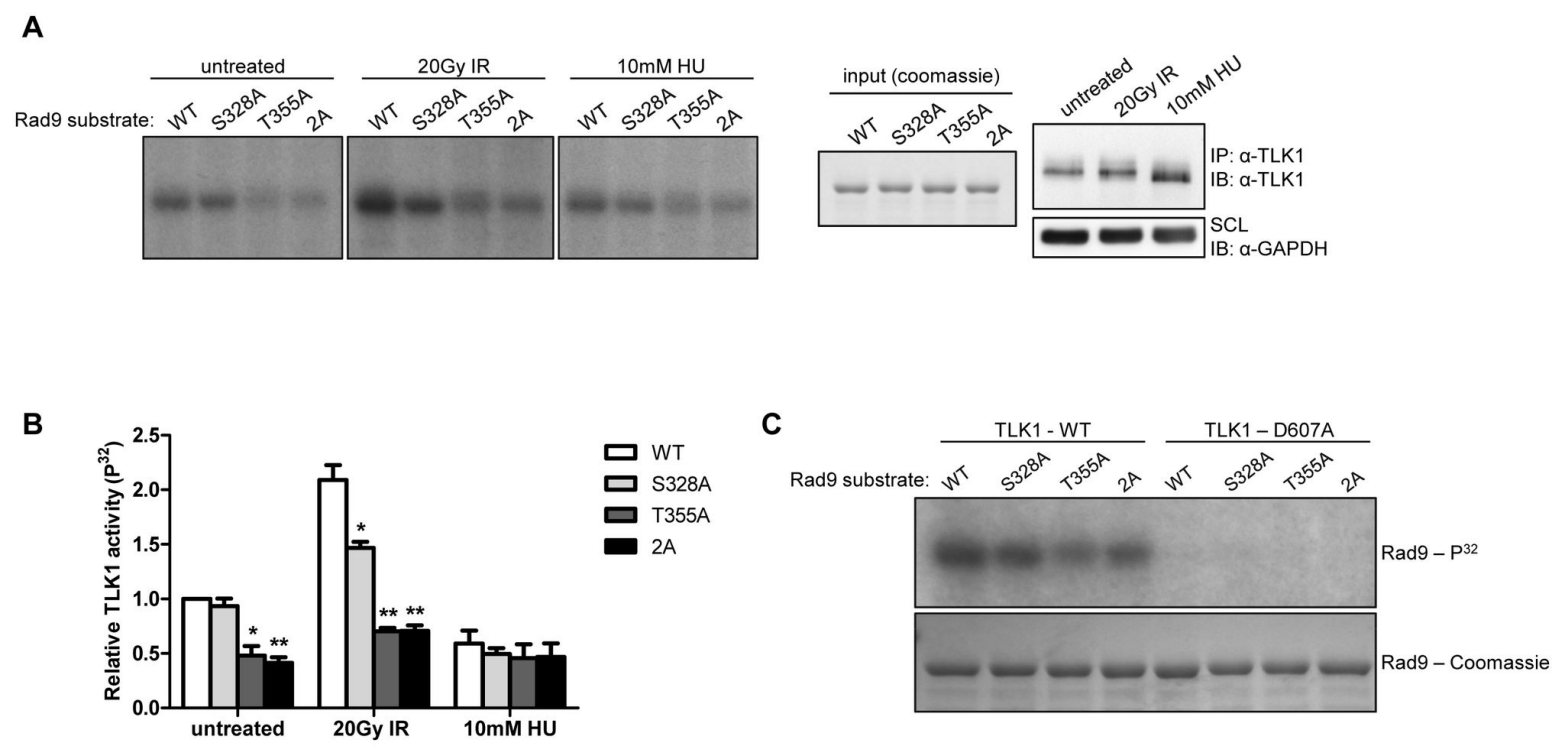
*In* *vitro* phosphorylation of full-length GST-Rad9 by TLK1. TLK1 was immunoprecipitated from HeLa cells and incubated with recombinant full-length GST-Rad9 bearing the indicated point-mutations. A. A representative autoradiograph (left panel) of a dried gel that was subsequently exposed to a phosphor-screen, quantitated, and corrected for background and Rad9 and TLK1 input (middle panel and right panel, respectively. 2A refers to a S328A/T355A double mutant. B. Phosphorylation was quantitated using a Storm 820 phospho-imager. Signal intensity was normalized against the amount of phosphorylation present in the untreated WT reaction. Error bars indicate the standard error of three independent experiments. Asterisks denote statistically significant differences compared to the level of phosphorylation of WT Rad9 within each treatment. p=0.034 (untreated T355A), p=0.004 (untreated 2A), p=0.0252 (IR – S328A), p=0.0067 (IR – T355A), p=0.0053 (IR – 2A). One asterisk denotes p ≤ 0.05. Two asterisks denote p ≤ 0.01. C. Similar to A, full-length GST-Rad9 constructs were incubated with WT and D607A myc-TLK1 immunoprecipitated from HeLa cells.

 Given the previous identification of Rad9 S328 as the residue modified by TLK1 [43], we sought to verify our *in vitro* kinase results using full-length Rad9 harboring the indicated point-mutations with N-terminal GST-fusion tags as substrates. Full-length WT Rad9 is phosphorylated by TLK1 immunoprecipitated from asynchronously growing HeLa cells (Figure 2A, left panel), and mutating S328 to alanine reduced phosphorylation only marginally. On the other hand, both a T355A mutant and a S328A/T355A double-mutant showed a reduction in phosphorylation of approximately 50% (p=0.0034 and p=0.004, respectively), and there was no discernible difference in phosphorylation levels between T355A and S328A/T355A, indicating that there is no additive effect when both residues are mutated. This suggests that TLK activity modifies Rad9 at T355 under asynchronous conditions. As with the C-terminal fragments, phosphorylation of each full-length Rad9 construct was enhanced when TLK1 was immunoprecipitated from cells that had been exposed to 20Gy IR. The S328A mutant displayed an approximate 30% reduction in phosphorylation compared to WT-Rad9 (p=0.0252), while phosphorylation of the T355A and S328A/T355A mutants were both reduced by approximately 65% (p=0.0067 and p=0.0053, respectively). Consistent with other work, and with Figure 1B, phosphorylation of each construct showed a trend towards lower levels following exposure to 10mM HU [42]. The assay was carried out in triplicate and quantified (Figure 2B) via phosphor-screen after correcting for substrate input and kinase input (Figure 2A, right panel), and normalized against the amount of signal present in WT-Rad9 incubated with TLK1 immunoprecipitated from untreated cells. As shown in Figure 2C, kinase-dead myc-TLK1 was unable to phosphorylate any of the full-length constructs. Taken together, these results show that there is a basal level of TLK-dependent phosphorylation at T355 of Rad9 *in vitro* that is increased after exposure to IR. Although T355 seems to be the preferred target, our data suggest that TLK1 also phosphorylates S328, and that this event may require prior phosphorylation at T355. Furthermore, the presence of latent phosphorylation in the 2A mutant (Figure 2A) indicates that TLK1 may phosphorylate other Rad9 residues *in vitro*, or that other kinases may have co-immunoprecipitated with TLK1 in trace quantities.

### The interaction between Rad9 and TLK1 requires chromatin-bound Rad9 and is enhanced at later stages of the checkpoint response

**Figure 3:**
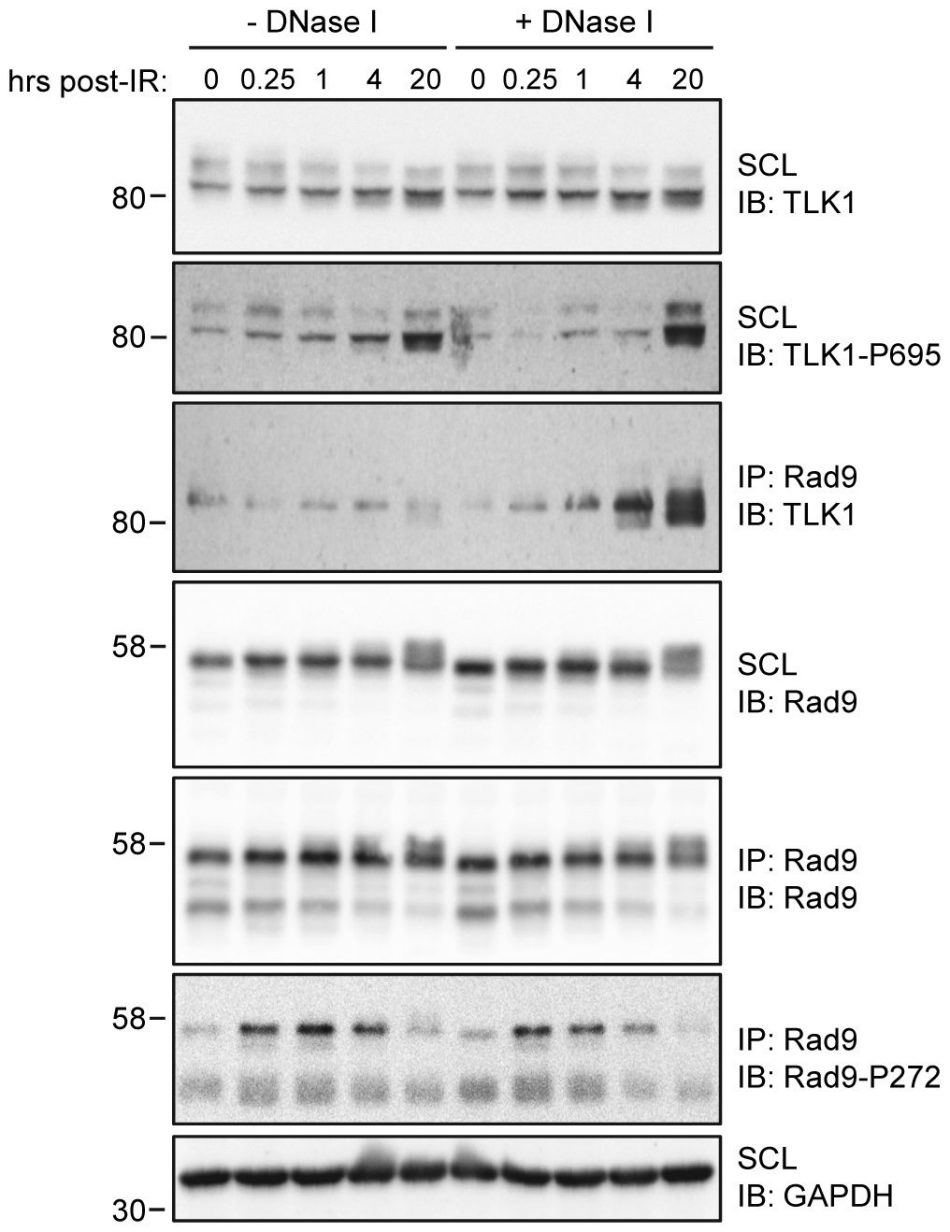
The association between Rad9 and TLK1 is enhanced late in the DNA damage response. HeLa cells were synchronized at the G1/S border with a single 2mM thymidine block for 18h, released for 2h, and then exposed to 10Gy IR and harvested at the indicated time points. Cells were lysed in NETN buffer in the presence or absence of DNase I. The resulting lysates were then subjected to immunoprecipitation using and antibody directed against Rad9. Immune complexes and soluble cell lysates were subjected to SDS-PAGE and immunoblotting, and probed with the indicated antibodies. SCL refers to soluble cell lysates, IB refers to immunoblot. Images are representative of three independent experiments.

Conventional co-immunoprecipitation techniques were employed to further characterize the interaction between TLK1 and Rad9. HeLa cells were synchronized at the G1/S barrier with a single thymidine block, after which they were released for 2hr. This was carried out to ensure a uniform cell cycle distribution upon subsequent exposure to IR. Cells were then exposed to 10Gy IR and harvested at progressive time-points in the presence or absence of DNase I, which serves to liberate chromatin-bound proteins that might otherwise precipitate during cell lysis [44–47]. As shown in the third panel of Figure 3, TLK1 and Rad9 were found to interact constitutively. Interestingly, this interaction was dramatically enhanced in later phases of the damage response (4hr and 20hr post-treatment) in lysates that were treated with DNase I, indicating that this interaction is damage-induced and dependent the association of Rad9 with chromatin. The levels of TLK1 present in the soluble cell lysate and immunoprecipitated Rad9 were consistent across different samples, indicating that the increased amount TLK1 co-immunoprecipitating with Rad9 was reflective of an enhanced degree of interaction. 

### Phosphorylation of Rad9 T355 fluctuates in undamaged HeLa cells as they progress through the cell cycle

**Figure 4:**
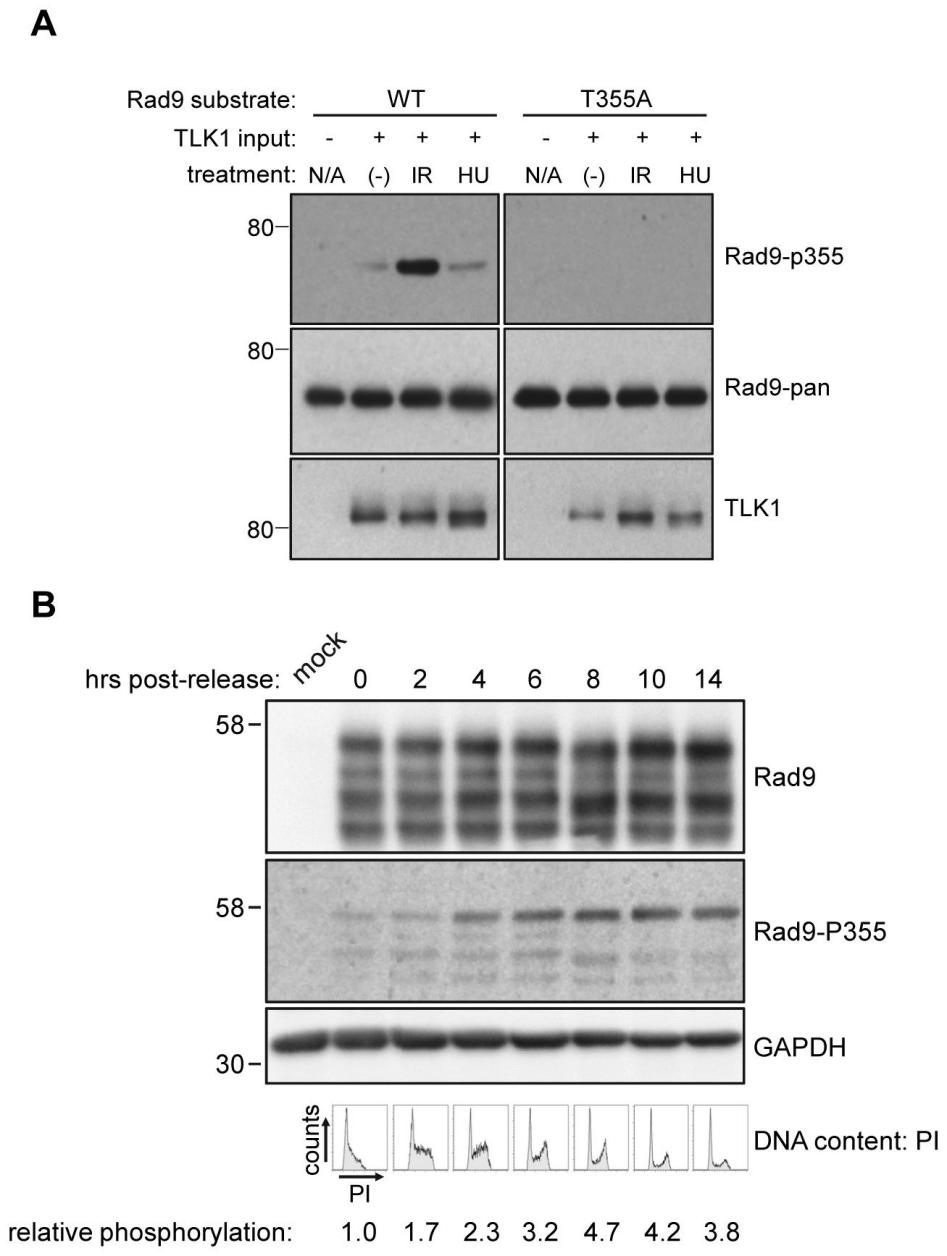
Phosphorylation at Rad9-T355 increases as cells enter mitosis. A. *In* *vitro* kinase assays were performed to verify the specificity of a novel Rad9 phospho-T355 antibody. TLK1 was immunoprecipitated from HeLa cells exposed to the indicated treatments and incubated with full-length WT or T355A GST-Rad9. Reactions were probed with the indicated antibodies. B. HeLa cells were transfected with wild-type Rad9, synchronized at the G1/S border, released and harvested at the indicated time points. Soluble cell lysates were equalized for protein content, subjected to SDS-PAGE and immunoblotting, and probed with the indicated antibodies. Cells from the same experiment were also analyzed for DNA content by flow cytometry after staining with PI. Immunoblots and histograms are representative of three independent experiments.

Given our data demonstrating phosphorylation of Rad9 T355 in undamaged cells, we sought to examine T355 phosphorylation levels in normally cycling. To this end, an antibody specific for phospho-T355 Rad9 was generated. To assess the specificity of this antibody, we carried out *in vitro* kinase assays by incubating WT and T355A full-length GST-Rad9 with immunoprecipitated TLK1, after which the reactions were fractionated via SDS-PAGE and probed with the indicated antibodies (Figure 4A). The phospho-T355 antibody was unable to detect recombinant Rad9 unless it has been incubated with TLK1 immune complexes, thus indicating that it is phosphorylation-specific, and not merely sequence specific. Next, HeLa cells were transfected with WT-Rad9 to facilitate antibody detection, and then synchronized at the G1/S border with a single-thymidine block, after which they were released and harvested at progressive time-points. Cells were concurrently stained with PI and analyzed for cell cycle distribution, and lysates were probed with the phospho-T355 antibody. Phosphorylation of T355 increased gradually as cell approached the G2/M boundary 6-8hr post-release (Figure 4B), after which it appeared to plateau as cells re-entered G1/S 10-14hr post-release. This indicates that T355 phosphorylation may be a factor in normal cell cycle regulation, in addition to the damage response. 

### Phosphorylation of Rad9 T355 in vivo drops rapidly and transiently in response to IR before returning to baseline levels

**Figure 5:**
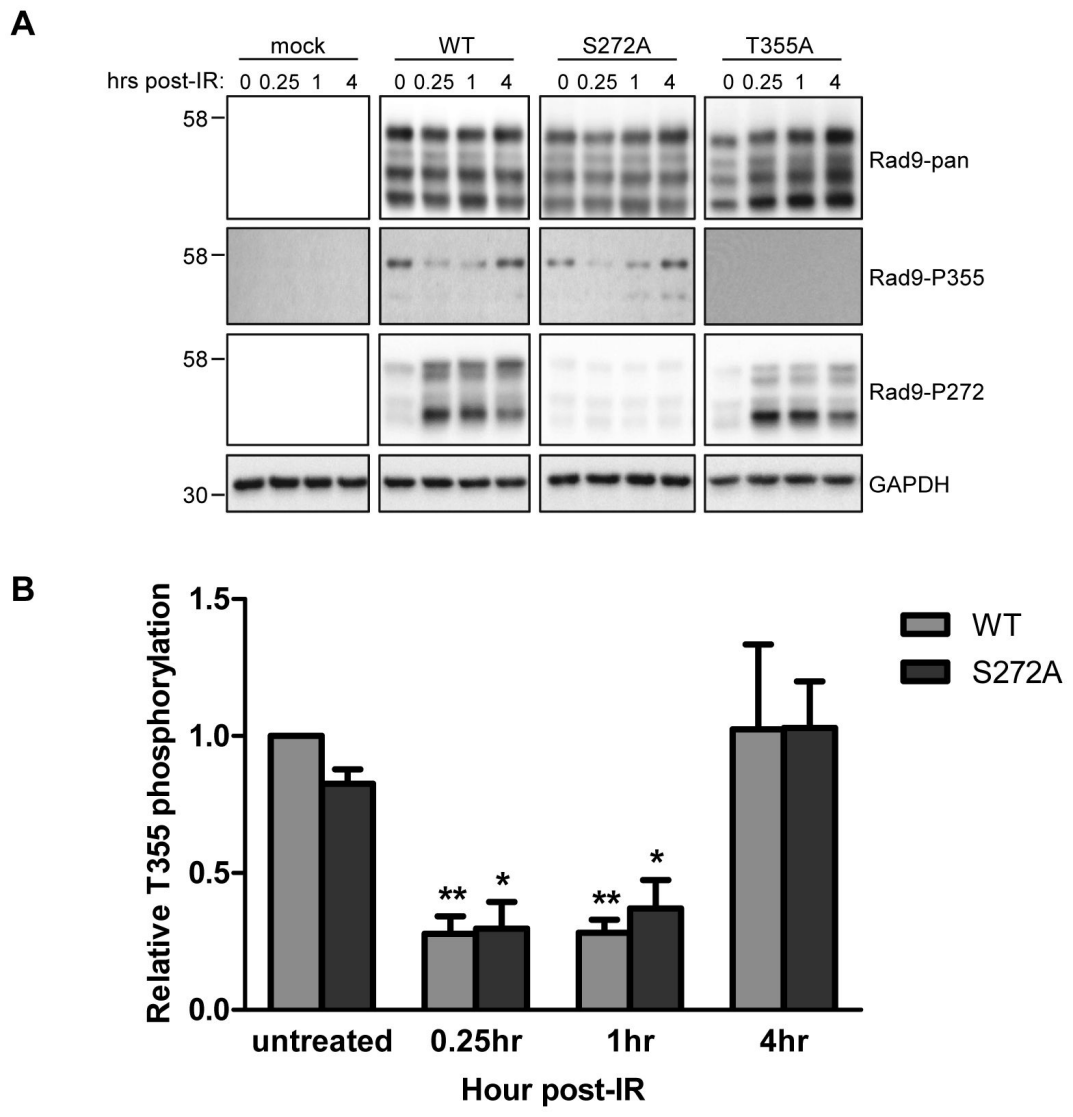
The phosphorylation status of Rad9-T355 fluctuates upon exposure to IR. A. HeLa cells were transfected with different pdDF-Rad9 constructs and synchronized at the G1/S border, after which they were exposed to 10Gy IR and harvested at the indicate time-points. Soluble cell lysates were subjected to SDS-PAGE and probed with the indicated antibodies. B. Phosphorylation at T355 was quantified by densitometry and corrected for the amount of total Rad9 and total protein. Error bars indicate the standard error of three independent experiments. Asterisks denote statistically significant differences compared to untreated cells for each construct. p=0.0038 (WT, 0.25hr post-IR), p=0.0023 (WT, 1hr post-IR), p=0.0204 (S272A, 0.25hr post-IR), p=0.0296 (S272A, 1hr post-IR). One asterisk denotes p ≤ 0.05. Two asterisks denote p ≤ 0.01.

Given our *in vitro* evidence for TLK-dependent Rad9 phosphorylation at T355, we set out to examine T355 phosphorylation levels throughout the damage response *in vivo*. HeLa cells were transfected with a panel of Rad9 mutants under control of the SR-α promoter and then synchronized with a single thymidine block for 18hr. The cells were released from thymidine and allowed to enter S-phase and then exposed to 10y IR and harvested at progressive time-points. The resultant lysates were then probed with the antibody specific for phospho-T355. The Rad9 constructs we employed were untagged to facilitate activation and DNA loading, but we can differentiate between endogenous and transfected Rad9 by varying exposure time [9]. Overexpressed Rad9 was used in this experiment, as the phospho-T355 antibody is not effective on endogenous levels of protein; faster migrating bands seen in some panels represent hypo-phosphorylated forms of Rad9 associated with overexpression (15,30). There was a baseline level of phosphorylation at T355 in untreated cells (Figure 5A) in both wild-type Rad9 and an S272A construct, a residue that is rapidly and transiently modified by ATM in response to damage [15,29,30,33], that was sharply reduced by 0.25hr post-treatment (p=0.0038 and p=0.0204, respectively). In the case of both constructs, phospho-T355 levels remained diminished at 1hr post-treatment (p=0.0023 and p=0.0296 respectively) before returning to baseline by 4hr. There was virtually no detectable signal with the T355A mutant, further indicating the specificity of the antibody. This assay was carried out in triplicate, and phosphorylation levels at T355 were quantified by densitometry and corrected for both the amount of total Rad9 and total protein present, after which the data was normalized against the level of T355 phosphorylation present in untreated WT Rad9 (Figure 5B). This modulation of phosphorylation levels bears similarity to the TLK activity profile, and suggests that the rapid loss of phosphorylation at T355 is an important initial step in the damage response. 

### TLK1 is required for normal cell cycle response and G2/M checkpoint activation in HeLa cells

**Figure 6:**
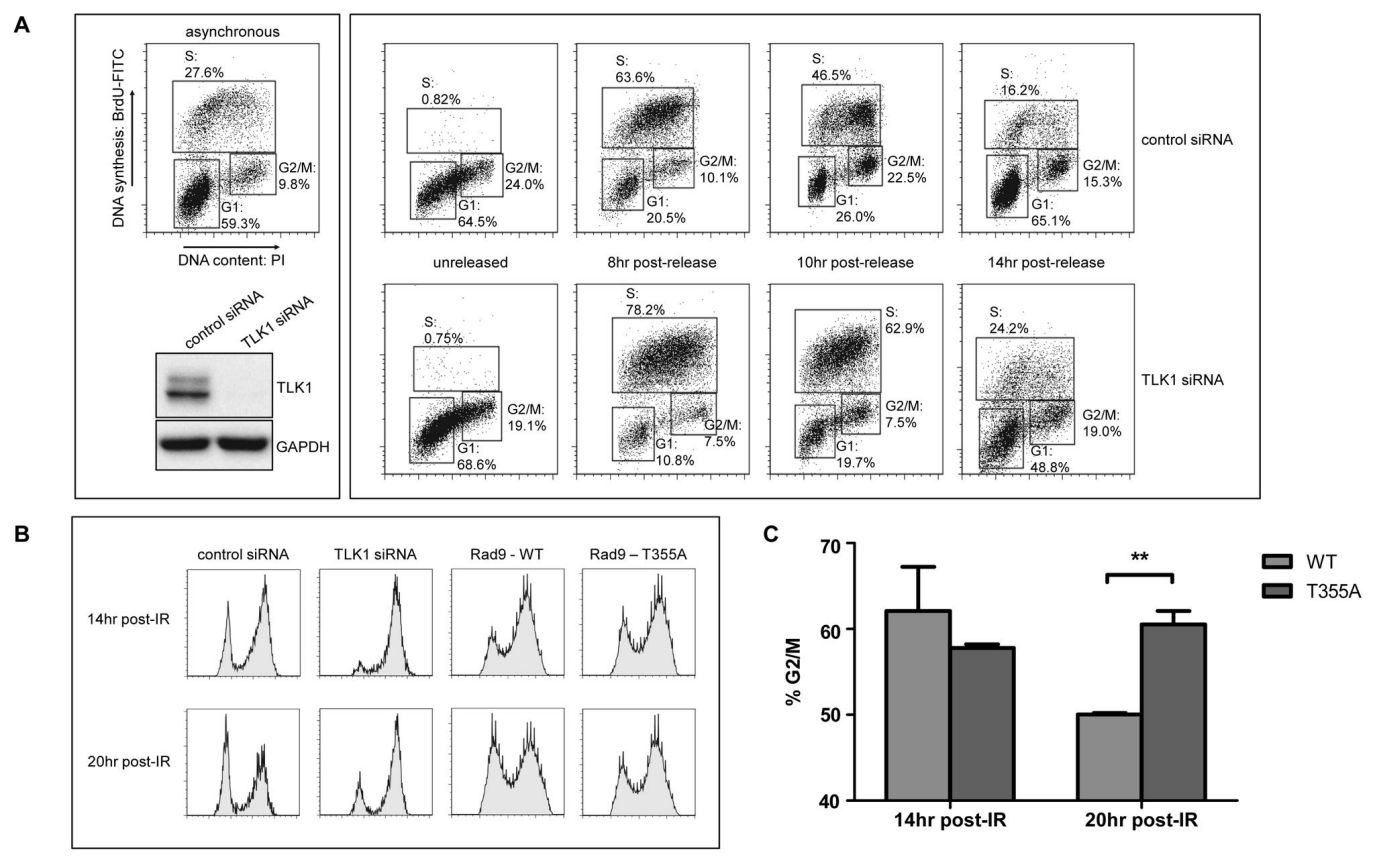
siRNA-mediated depletion of TLK1 delays cell cycle progression and causes prolonged G2/M arrest after exposure to IR. A. Cells were transfected with an siRNA directed against TLK1 and then synchronized at the G1/S, after which they were harvested at the indicated time-points post-release and stained with PI and a FITC-conjugated α-BrdU antibody. B. HeLa cells were transfected with an siRNA directed against TLK1 or plasmids encoding WT and T355A Rad9. Cells were synchronized at the G1/S border, released for 2hr, and then exposed to 10Gy IR and harvested at the indicated time-points post-treatment. Cell cycle distribution was analyzed by flow cytometry. C. The proportion of cells transfected with WT or T355A Rad9 in G2/M at 14hr and 20hr after exposure to 10Gy IR. Error bars indicate the standard error from three independent experiments. The asterisks denote a statistically significant difference between the G2/M populations comparing WT to T355A transfected cells (p=0.0092).

Our next aim was to examine the role of TLK1 in cell cycle progression and checkpoint response by depleting TLK1 expression via siRNA-mediated knockdown. To accomplish this, HeLa cells were transiently transfected with an siRNA directed specifically against TLK1 (and not TLK2), and cell cycle distribution was monitored by pulse-labeling cells with BrdU, after which they were dual-stained with PI and a FITC-conjugated antibody directed against BrdU. When HeLa cells were synchronized at the G1/S border with a single thymidine block and released, cells lacking TLK1 progressed into S-phase normally but appeared to accumulate there, and subsequently failed to transition efficiently into mitosis and then back to G1 when compared to cells transfected with a non-silencing control siRNA (Figure 6A). 

 HeLa cells depleted for TLK1 also display prolonged G2/M checkpoint activation in response to IR. As expected based on previous work in our lab and others, HeLa cells transfected with a non-silencing control siRNA exhibit G2/M checkpoint activation 14hr after exposure to 10Gy IR (Figure 6B), and the increased proportion of cells in G1 at 20hr post-IR indicates that a subpopulation of G2/M arrested cells is able to escape the checkpoint [15]. In contrast, silencing of TLK1 resulted in a pronounced and prolonged G2/M arrest in response to IR. A much larger proportion of cells were arrested in G2/M at both 14hr and 20hr post-IR upon TLK1 depletion, indicating that TLK activity is required for checkpoint release in HeLa cells. It appears that TLK1 plays a role in enabling the exit of G2/M-arrested cells from the damage-induced checkpoint and re-entry into G1. 

Finally, we wanted to establish if there is a connection between the checkpoint phenotype induced by TLK1 depletion and reduced phosphorylation of Rad9 at T355. HeLa cells transiently over-expressing WT or T355A Rad9 were synchronized at the G1/S border and released for 2hr, after which they were exposed to 10Gy IR, harvested 14hr and 20hr after treatment, and analyzed for cell cycle distribution by flow cytometry. Indirect Rad9 immunoflourescence indicates that transfection efficiency was approximately 50%. Cells transfected with either WT or T355A Rad9 both exhibited G2/M checkpoint activation 14hr after treatment (Figure 6B). By 20hr post-treatment, a population of WT-transfected cells had exited the G2/M checkpoint and re-entered G1. Conversely, cells that over-expressed T355A were still predominantly arrested in G2/M compared to WT (Figure 6C, p=0.0092). Thus, over-expression of Rad9-T355A mimics TLK1 depletion in that cells are less efficient at exiting the damage-induced G2/M checkpoint. 

## Discussion

 In this study we have characterized the relationship between Rad9 and TLK activity, and demonstrate that this interaction is a functional component of the checkpoint response. We base this on four principle findings: 1) immunoprecipitated TLK1 phosphorylates Rad9 *in vitro* preferentially at T355, and kinase-dead myc-TLK1 is unable to phosphorylate Rad9; 2) *in vivo* phosphorylation of Rad9 at T355 fluctuates in a damage-dependent manner consistent with it being a TLK substrate; 3) knockdown of TLK1 leads to delayed cell cycle progression and prolonged accumulation of HeLa cells in G2/M following irradiation; and 4) over-expression of a Rad9 mutant construct that cannot be phosphorylated at T355 mimics the checkpoint defect of TLK1 depletion. 

 The data presented here and elsewhere [48] strongly indicates that TLK1 regulates Rad9 function. A recent study showed that TLK1 and Rad9 physically interact *in vitro* and that Rad9 is substrate for TLK1 [43]. However, the previous report indicated that TLK1 phosphorylates Rad9 at S328, whereas our data shows that T355 is the preferred residue (Figure 1B & Figure 2A). There are a few potential explanations for this discrepancy. The previous study employed recombinant N-terminally truncated TLK1, representing the TLK1 splice variant TLK1B, in their kinase assays while we used full-length TLK1 immunoprecipitated from HeLa cells. It is possible that immunoprecipitated full-length TLK1 preserves post-translational modifications and binding partners that affect TLK activity in a way that bacterially-expressed and truncated TLK1 does not, and thus is a better physiological reflection of TLK function. We also demonstrated that immunoprecipitated kinase-dead, myc-tagged TLK1 is unable to phosphorylate Rad9 *in vitro*, indicating that the phosphorylation we are detecting in TLK1 immune complexes is TLK1-direct (Figure 1D). In addition, they identified the region of Rad9 subject to TLK1-dependent modification by mass-spectrometry of tryptic peptides and focused specifically on the peptide containing S328. It should be noted that while our data indicates that T355 is the preferred target for TLK1 *in vitro*, it also indicates that TLK1 is capable of modifying S328. In fact, the reduction in phosphorylation found in the full length Rad9 S328A mutant compared to the more dramatic change seen in T355A (Figure 2) may suggest that phosphorylation at T355 is a prerequisite for S328 phosphorylation. Thus, loss of phosphorylation at T355 may in and of itself reduce S328 phosphorylation. Given that there is very little S328 phosphorylation in the fragments we employed that correspond Rad9’s C-terminal tail (Figure 1A), it may also be the case that TLK-dependent phosphorylation of S328 requires the full context of the Rad9 protein. 

 Previous work has shown that peak TLK activity with regards to ASF1A occurs during S-phase, and that TLK1, in particular, is inactivated rapidly in response to damage in an ATM-Chk1-dependent manner before returning to baseline levels [36,41,42]. Our *in vivo* data are consistent with Rad9 being a substrate for TLK1, as we show that there is a basal level of Rad9 T355 phosphorylation in unperturbed cells that is rapidly diminished after exposure to IR, and returns to baseline later in the damage response (Figure 5). This is suggestive of a role for TLK-dependent Rad9 phosphorylation in normally cycling cells and in checkpoint recovery. TLK-dependent phosphorylation of Rad9 at S328 and T355 may be a signal for re-setting Rad9 function and disengaging the 9-1-1 complex from DNA such that cells can terminate checkpoint signaling and resume cell cycle progression once DNA lesions have been repaired [49–51]. Previous work from our lab indicates that Cdc2 phosphorylates Rad9 at multiple residues *in vitro* and *in vivo*, and that T355 may be one of the potential targets [15]. Thus, both Cdc2 and TLK1 may target Rad9 and promote cell cycle turnover and checkpoint exit through redundant mechanisms. Our results showing that peak T355 phosphorylation in unperturbed cells occurs as they enter mitosis following synchronization at the G1/S border (Figure 4) further supports the possibility that TLK-dependent regulation of Rad9 plays a role cell cycle turnover and checkpoint recovery. This likely explains why TLK1 immunoprecipitated from HeLa cells exposed to IR displayed greater activity with regards to Rad9 than those growing asynchronously. An asynchronous population contains cells from all phases of the cell cycle, whereas those that have been exposed to IR are essentially synchronized as they enter the G2/M checkpoint and subsequent checkpoint recovery.

 We have shown, using siRNA-mediated knockdown, that TLK1 is required for normal progression through S-phase and reduction of TLK1 results in prolonged G2/M checkpoint activation in HeLa cells (Figure 6). We can not formally disprove the possibility that TLK1 knockdown leads to a mitotic arrest, rather than G2-M arrest, but feel this is unlikely due to the established activity profile of TLK1 (peak in S-phase, inhibited by damage early), as well as the S-phase defect seen in Figure 6A. The depletion of TLK1 may cause a decrease in the amount of phosphorylated ASF1A which could leave it vulnerable to proteasomal degradation [40,42]. This in turn could impede nucleosome re-assembly during replication [38,52] and may explain the delay in S-phase progression. Conversely, or concomitantly, a reduction in the amount of Rad9 phosphorylation at T355 may prevent Rad9 and the 9-1-1 complex from disengaging from chromatin and thus delay preparation for the next cell cycle. Future studies will address this possibility.

 TLK1 depletion resulted in a prolonged accumulation of cells in G2/M following irradiation (Figure 6B). Prolonged G2/M accumulation following damage is a common phenotype among cells with defective S-phase checkpoints [53–55] which may represent a compensatory mechanism to allow cells more time to repair aberrantly replicated DNA. However, neither TLK1 nor TLK2 have been implicated in the S-phase checkpoint; in fact, the available evidence demonstrates that they are initially inhibited in response to damage. A potential explanation may be that TLK activity is required in the subset of cells that are able to exit the G2/M checkpoint, complete mitosis and re-enter G1. This is consistent with the TLK activity profile that we and other groups have shown {[41,42], (Figure 5B&C)}. Furthermore, our data showing that overexpression of Rad9 T355A also causes a prolonged G2/M checkpoint (Figure 6B&C) suggests that the return of TLK-dependent Rad9 phosphorylation at later stages following IR may facilitate the cessation of checkpoint signaling and alleviate cell cycle arrest, thus allowing cells to resume cell cycle progression. The specifics of how this would occur remain unclear, although we speculate that phosphorylation of Rad9 at T355 may inhibit loading of the 9-1-1 complex, and promote disengagement from chromatin and subsequent checkpoint release. Alternatively, or potentially cooperatively, the interaction between TLK1 and Rad9 may extend beyond phosphorylation. The IR-enhanced association between TLK1 and Rad9 is more persistent than a typical kinase/substrate interaction (Figure 3), and a previous study suggested that TLK1 could promote chromatin assembly independent of its kinase activity, and that *in vitro* binding of Rad9 with TLK1 was competitive with the histone chaperone ASF1A [43]. Therefore, in addition to phosphorylation, TLK1 may play a direct role in disengagement of the 9-1-1 complex from DNA and promote re-assembly of chromatin post-repair. Further studies are certainly needed to clarify the precise mechanism of the interaction between Rad9 and TLK1. 

 Taken together, these results point to the existence of a potential Rad9-Chk1-TLK-Rad9 feedback loop that regulates checkpoint function. The requirement of Rad9 for ATR-mediated Chk1 activation has been well-established [17,29,56], as has the transient negative regulation of TLK1 by Chk1 [41,42]. We speculate that in the early stages of the damage response the 9-1-1 complex facilitates Chk1 activation, which in turn inhibits TLK activity and leads to a reduction of Rad9 phosphorylation levels at T355, thus maintaining the checkpoint. TLK activity returns as cells enter the checkpoint recovery phase, thus increasing Rad9 T355 phosphorylation and alleviating the checkpoint-induced arrest. 

 The non-PCNA-like C-terminal tail of Rad9 represents a likely regulatory domain for the 9-1-1 complex in checkpoint signaling [15,17,19,30,57], although the complex nature of phosphorylation in this region has made it challenging to identify the residues modified and their physiological significance. Our data strongly implicate that TLK activity regulates Rad9 and thus the 9-1-1 complex during unperturbed cell cycle progression and in the recovery stage of the G2/M checkpoint. We speculate that TLK-dependent Rad9 phosphorylation plays a role in re-setting the 9-1-1 complex as cells complete mitosis and re-enter G1, and in checkpoint recovery as cells have repaired damaged DNA are able to resume cell cycle progression. Improper regulation of this process caused by increased TLK activity (or deficient TLK inhibition) could promote cell survival after DNA damage, which in turn could lead to increased mutation rates, genomic instability and radioresistance. Several human cancers have been identified that feature mutations in the kinase domain of TLK1 [58–62], and high TLK1 expression levels correlate with radioresistance [63,64] thus raising the possibility that TLK1 is an oncogene and potential therapeutic target.
